# Efficient Integration of Coupled Electrical-Chemical Systems in Multiscale Neuronal Simulations

**DOI:** 10.3389/fncom.2016.00097

**Published:** 2016-09-12

**Authors:** Ekaterina Brocke, Upinder S. Bhalla, Mikael Djurfeldt, Jeanette Hellgren Kotaleski, Michael Hanke

**Affiliations:** ^1^Science for Life Laboratory, Computational Science and Technology, School of Computer Science and Communication, KTH Royal Institute of TechnologyStockholm, Sweden; ^2^National Centre for Biological SciencesBangalore, India; ^3^Manipal UniversityManipal, India; ^4^PDC Center for High-Performance Computing, KTH Royal Institute of TechnologyStockholm, Sweden; ^5^International Neuroinformatics Coordinating Facility, Karolinska InstituteStockholm, Sweden; ^6^Science for Life Laboratory, Department of Numerical Analysis and Computer Science, Stockholm UniversityStockholm, Sweden; ^7^Department of Neuroscience, Karolinska InstituteStockholm, Sweden; ^8^Department of Mathematics, School of Engineering Sciences, KTH Royal Institute of TechnologyStockholm, Sweden

**Keywords:** multiscale modeling, multiscale simulation, co-simulation, coupled system, adaptive time step integration, backward differentiation formula, parallel numerical integration, coupled integration

## Abstract

Multiscale modeling and simulations in neuroscience is gaining scientific attention due to its growing importance and unexplored capabilities. For instance, it can help to acquire better understanding of biological phenomena that have important features at multiple scales of time and space. This includes synaptic plasticity, memory formation and modulation, homeostasis. There are several ways to organize multiscale simulations depending on the scientific problem and the system to be modeled. One of the possibilities is to simulate different components of a multiscale system simultaneously and exchange data when required. The latter may become a challenging task for several reasons. First, the components of a multiscale system usually span different spatial and temporal scales, such that rigorous analysis of possible coupling solutions is required. Then, the components can be defined by different mathematical formalisms. For certain classes of problems a number of coupling mechanisms have been proposed and successfully used. However, a strict mathematical theory is missing in many cases. Recent work in the field has not so far investigated artifacts that may arise during coupled integration of different approximation methods. Moreover, in neuroscience, the coupling of widely used numerical fixed step size solvers may lead to unexpected inefficiency. In this paper we address the question of possible numerical artifacts that can arise during the integration of a coupled system. We develop an efficient strategy to couple the components comprising a multiscale test problem in neuroscience. We introduce an efficient coupling method based on the second-order backward differentiation formula (BDF2) numerical approximation. The method uses an adaptive step size integration with an error estimation proposed by Skelboe (2000). The method shows a significant advantage over conventional fixed step size solvers used in neuroscience for similar problems. We explore different coupling strategies that define the organization of computations between system components. We study the importance of an appropriate approximation of exchanged variables during the simulation. The analysis shows a substantial impact of these aspects on the solution accuracy in the application to our multiscale neuroscientific test problem. We believe that the ideas presented in the paper may essentially contribute to the development of a robust and efficient framework for multiscale brain modeling and simulations in neuroscience.

## 1. Introduction

The concept of multiscale modeling is used in many fields such as meteorology (Shukla, 2009; Kurowski et al., 2013), cardiac physiology (Hernández et al., 2011) and neuroscience (Bhalla, 2014). It refers to the style of modeling in which different models, possibly described by different physical formalisms and acting on different temporal and spatial scales, are used simultaneously in order to study important features of a complex phenomenon at multiple levels of organization (Djurfeldt and Lansner, 2007).

An idea to study how one level of neural organization influences another has become an important trend in neuroscience. For example, Bhalla (2011) explores cross-scale interactions between cellular and subcellular levels in the context of homeostasis and synaptic plasticity. This multiscale model proposes a pruning mechanism for weak synapses during cellular excitability. Another study by Mattioni and Le Novère (2013) presents an integration of electrical and biochemical processes in a model of a Medium Spiny Neuron (MSN). In particular, the influence of different input patterns on membrane excitability and the mechanism of inter-spine synaptic plasticity is discussed.

This paper focuses on the numerical aspects of different coupling strategies. The goal of the paper is to present an efficient method applicable to a wide class of problems where electrical activity and chemical signals must be solved together in the cell. This refers to almost all classes of excitable cells such as muscle fibers and nerve cells or neurons. In the neuronal context, the class of problems includes synaptic plasticity, excitability homeostasis, development and other specific functions that involve such multiscale cellular events. Here, we present a method that allows us to bridge subcellular and cellular level models described by ODEs in an accurate and efficient way.

How should the numerical solution of a multiscale system be arranged in practice? Different components of the system may need to be solved using different numerical methods. This can be the case if the components are described by different physical formalisms, for example one employs ordinary differential equations (ODEs) and the other is stochastically formulated. Even in the case when a multiscale system is formulated by a single formalism it may become advantageous to treat different components separately, both with regard to their description and with regard to computing the solution. If different components of a system are treated as independently as possible, computations can be arranged to take advantage of modularity and parallelism.

Kübler and Schiehlen (2000) propose that a complex engineering system can be decomposed into modules at three levels of description: the physical, mathematical and behavioral levels. The components of a system can be integrated at the mathematical level and then solved using a single numerical solver. Here, we rather focus on coupling at the behavioral level—the level of signals in the computed solution. In particular, we are interested in coupling different numerical solvers in a theoretically proper and efficient way.

There are a few considerations that have to be taken into account while coupling the components at the behavioral level. First, a minimal set of signals which need to be communicated between system components has to be defined. Then, an organization between system components play a crucial role in an accurate and efficient integration of the coupled system. Mattioni and Le Novère (2013) proposed an event-driven algorithm where the exchange of the scaled variables have to be communicated each time the event happens. The algorithm showed better performance results in comparison with the time-driven algorithm where the communication of exchanged variables has to be performed at regular time intervals. However none of the recent studies give a theoretical background for the proposed coupling strategy.

One of the problems which can occur when coupling system components is numerical instability (Arnold and Günther, 2001). Numerical methods must ensure convergence of the discrete system. Even if a coupled integration is convergent, a proper choice of the step size still has to be made in order to guarantee numerical stability. It is important to keep in mind that the numerical stability of a coupled integration is not guaranteed by the stability of an independent integration of the system components (see Supplementary Instability Example). At the same time, it is crucial to perform integration in an efficient way while keeping the accuracy within desired bounds. Finally, the complexity increases when system components are described by different physical formalisms (Alfonsi et al., 2005; Rüdiger et al., 2007; Brandi et al., 2011). For example, chemical interactions can be described either in a deterministic or in a stochastic way possibly accompanied by diffusion processes.

We begin with a general introduction to the modeling at different levels within the scope of interest. In particular, in Section 2.1, we give an overview of some widely used numerical discretization methods in Neuroscience. Section 2.2 covers different numerical aspects of a coupled integration such as efficiency, order of accuracy and numerical stability. In Section 2.3, we introduce an algorithm for an adaptive control of the step size in a coupled integration. In Section 2.4, we present possible organization strategies in the system composed of multiple components. Section 2.6 describes the multiscale test model used for our analysis: the dynamics, communication signals between the components and mathematical formulation. The details of implementation and evaluation can be found in Sections 2.7 and 2.8, respectively. In Sections 3 and 4, we present and discuss the results.

## 2. Materials and methods

### 2.1. Modeling at different levels

Here, we focus on cellular and subcellular (molecular) levels of neuronal organization. On the cellular level the object of study is typically electrical properties of a cell. The cell with its complex arborizations is usually represented by a cable split into a number of compartments. Modelers then employ compartmental modeling (Rall, 1964) to describe the neural processes where the dynamics of each compartment is defined by a system of ordinary differential equations (ODEs). Finally, by applying the Hodgkin and Huxley formalism to define the currents (Hodgkin and Huxley, 1952) a nonlinear system of ODEs has to be solved. This approach is the basis of most simulators that take neural morphology into account [e.g., NEURON (Hines, 1989), GENESIS (Wilson et al., 1990), MOOSE (Dudani et al., 2009)].

On a molecular level the interaction of biochemical signaling pathways is of particular interest. A signaling pathway is usually considered as a set of reactions between the molecules that operate on a subcellular level (Bhalla, 1998). Chemical rate theory is normally applied to describe the chemical kinetics of a signaling pathway. One of the traditional ways to model the kinetics is by viewing the system of reactions as deterministic. Then the chemical species are typically modeled as concentrations that evolve over time. These models are described by a system of nonlinear ODEs.

The complexity of the systems requires numerical computations. Simulation packages offer the user a choice between different numerical integration methods. The method choice is usually dependent on the properties of a system. For example, if the system of ODEs is *stiff* and an explicit numerical method has been chosen, the step size of the discretization is limited by stability and not by accuracy. Thus an efficient numerical integration rather requires *implicit* methods. Implicit methods often allow the simulation to be discretized with larger time steps due to good stability properties.

Fixed step size numerical methods, such as the modified Crank-Nicholson method and the classical Runge-Kutta method, are typically applied to solve systems of current study in neuroscience. The choice of the discretization time step is then made by running the simulation with different step sizes and comparing the computation cost vs. solution accuracy.

The Crank-Nicholson (CN) method with a staggered time step approach is widely used for solving branched nerve equations in neuroscience (Hines, 1984). The proposed approach allows the user to obtain a solution in an accurate and efficient way. The Crank-Nicholson method is an implicit method and can be used for stiff systems. However little is known about stability properties while working on a staggered grid (see Supplementary Numerical Methods).

For the models defined on a subcellular level, the Classical Runge-Kutta (RK4) numerical approximation method got its wide application among computational neuroscientists. It is an explicit numerical method with a bounded stability domain and therefore it is not suitable for stiff problems. A mathematical formulation of the method can be found in Supplementary Numerical Methods.

While both the CN on a staggered grid and the RK4 methods provide efficient means for simulating the models on the appropriate levels, the coupling of these methods poses additional questions.

### 2.2. Numerical considerations in a coupled integration

#### 2.2.1. Efficiency

Multiscale systems are usually composed of components acting on different timescales. For example, the timescale of a single spike is of the order of a few milliseconds. However, simulations normally run for many seconds in order to observe the effects at a biochemical level. The gap between timescales demands an efficient integration strategy. We suspect that adaptive integrators can be more efficient or require less time for a given degree of accuracy. Both the CN on a staggered grid and the RK4 methods are normally applied on a fixed step size grid. Neither provide an error estimation that could be used together with a step size control mechanism.

#### 2.2.2. Order of accuracy

Little is known about the error propagation for the CN on a staggered grid with RK4 coupled integration. The order of the numerical method quantifies the global error behavior with respect to the step size. We know that the CN on a staggered grid method is second-order accurate and the RK4 method is of the order four while the order of the coupled integration still has to be studied.

#### 2.2.3. Numerical stability

Numerical properties, such as numerical stability, of a numerical algorithm applied to each system component independently may not hold in a coupled integration. For example, the Backward Euler method applied to a stable system may lead to an amplified oscillating behavior of the system when solved in a coupled manner as shown in Supplementary Instability Example. This may happen due to the different component properties, such as stiffness, that may arise in a coupled system simulation. Our multiscale system is often stiff due to the rapid changes in the electrical component. Thus, to be on the safe side we aim to avoid using methods not suitable for stiff problems.

### 2.3. Numerical algorithm

#### 2.3.1. Backward differentiation formula

Considering the thoughts above, we are interested in another family of implicit methods, Backward Differentiation Formula (BDF). In particular, the second-order BDF (BDF2) method has gained a large recognition in its application to stiff differential equations and Differential Algebraic Equations (DAEs). Moreover, Skelboe (2000) provides stability analysis and error estimates within a coupled numerical integration strategy. In Skelboe (2000) refers to the method as to the decoupled BDF2 formula. According to the algorithm a given system has to be partitioned into loosely coupled subsystems first and then the decoupled formula is applied to integrate the system. Here, the subsystems to be coupled are given beforehand. A mathematical formulation of the method is given in Supplementary Numerical Methods.

#### 2.3.2. Adaptive step size controller

The aim of the adaptive step size controller is to reduce the computational cost of the simulation while keeping the local error within acceptable bounds. The reduced computational cost is mainly achieved by a reduced number of time steps at which a solution is approximated. In particular, the step size controller aims at guaranteeing Equation (1) for an as large as possible step size *h*, where |ϵ_*j*_| is the local discretization error and the parameter tolerance *TOL* is a bound on the local error.

(1) $$\left|\epsilon_{j}\right|\leq TOL$$

We implement an adaptive control of the step size shown in Algorithm 1. It calculates a local discretization error |[ϵ_*j*_]| of the taken step *h*_*j*_ (line #8). If the calculated quality is good enough (line #9), an optimal step size is calculated and then used as a predictor for the next step size *h*_*j*+1_ (line #13). Otherwise, the current step size *h*_*j*_ is recalculated (line #16) and the step has to be done once again.

**Algorithm 1:** Step size controller.

1: $h_{0}={\bar{h}}_{0}$ % Choose initial step size prediction ${\bar{h}}_{0}$
2: *j* = 0 % Initiate the iteration index
3: Δ_*t*_ = {*t*_0_} % Initiate the time set
4: *x*_Δ_(*t*_0_) = *x*_0_ % Initiate the solution set
5: **while** *t*_*j*_ < *T* **do** % Within the simulation time *T* do:
6: *t* = *t*_*j*_ + *h*_*j*_
7: $x=\Psi^{t,t_{j}}x_{\Delta }\left(t_{j}\right)$ % Advance the solution from *t*_*j*_ to *t*
8: compute the error estimate |[ϵ_*j*_]|
9: **if** |[ϵ_*j*_]| ≤ *TOL* **then** % Step is accepted
10: *t*_*j*+1_ = *t*
11: Δ_*t*_ = Δ_*t*_∪{*t*_*j*+1_} % Update the time set
12: *x*_Δ_(*t*_*j*+1_) = *x* % Update the solution set
13: *h*_*j*+1_ = min(*qh*_*j*_, *h*_*max*_, $H211b\left(h_{j},\vec{\left|\left[\epsilon \right]\right|}\right),$ (*T*−*t*_*j*+1_)) % Calculate an optimal time step *h*_*j*+1_ % using Söderlind H211b controller (Söderlind and Wang, 2006)
14: *j* = *j*+1
15: **else** % Step is rejected
16: *h*_*j*_ = min(*qh*_*j*_, *h*_*max*_, $\sqrt[{p+1}]{\frac{\rho TOL}{\left|\left[\epsilon_{j}\right]\right|}}h_{j})$ % Adjust the current time step *h*_*j*_ % using a P-controller (Deuflhard and Bornemann, 2002, pp. 197ff)
17: **end if**
18: **end while**

Since the quantity of the local discretization error |*e*_*j*_| cannot be determined *exactly*, the notion of some *computable estimate* |*e*_*j*_| ≈ |[ϵ_*j*_]| was introduced by Deuflhard and Bornemann (2002).

In Algorithm 1 we apply two controllers depending on the computed local discretization error value to predict an optimal time step, a so-called H211b controller proposed by Söderlind and Wang (2006) and a P-controller used by Deuflhard and Bornemann (2002, pp. 197ff). The optimal time step is also bounded by *qh*_*j*_ (*q* > 1) and *h*_*max*_ terms for the situation when the error estimation value becomes or is close to zero (line #13 and line #16).

In order to implement Algorithm 1 an error estimation mechanism (line #8) should be provided.

#### 2.3.3. Estimate of the local discretization error

The error estimation is method dependent. Below we describe the error estimation for the BDF2 method based on a predictor-corrector algorithm. The difference of the discrete evolutions given by Equation (2) represents an estimate of the local error |[ϵ]|:

(2) $$|\left[\epsilon_{j}\right]|=\left\|\Psi^{t+h,t}x-{\hat{\Psi }}^{t+h,t}x\right\|,$$

where the prediction step taken by the discrete evolution ${\hat{\Psi }}^{t+h,t}$ calculates a rough approximation of the solution and the corrector step taken by Ψ^*t*+*h, t*^ refines the initial approximation.

Often, the tolerance *TOL* is set as a combination of a relative tolerance, *relTOL*, and an absolute tolerance, *absTOL*. Then, considering Equations (1) and (2) we can write:

(3) $$\left\|\Psi^{t+h,t}x-{\hat{\Psi }}^{t+h,t}x\right\|\leq relTOL\;\cdot \;\left\|\Psi^{t+h,t}x\right\|+absTOL$$

From a practical point of view, it is more appropriate to use a similar requirement component-wise. By rearranging Equation (3) we obtain the error control quantity |[ϵ]| with the bound equal to one:

(4) $$\left|\left[\epsilon \right]\right|=\underset{i}{\max}\frac{\left|\Psi^{t+h,t}x_{i}-{\hat{\Psi }}^{t+h,t}x_{i}\right|}{relTOL\;\cdot \;\left|\Psi^{t+h,t}x_{i}\right|+absTOL_{i}}\leq 1$$

where *i* is the index of the integrated variable in the solution vector.

The best efficiency is usually achieved if both the predictor and the corrector are of the same order (Sjö, 1999). Furthermore polynomial interpolation formulas are preferred as predictors in connection with stiff problems (Skelboe, 2000). The prediction step in Equation (4) is taken by the discrete evolution of the second-order polynomial described in Section 2.5, and the corrector step by the BDF2 method.

### 2.4. Organization of system components in a coupled integration

In the coupled integration the components of a system are solved independently on time windows [*T*_*n*_, *T*_*n*+1_]. Then information is exchanged at communication points 0 = *T*_0_ < ⋯ < *T*_*n*_ < *T*_*n*+1_ < … . Different aspects of integration are considered in the literature: the use of multiple discretization step sizes (*multirate* methods), the coupling of different numerical methods (*multi-method* integration) and different types of organization of computations between the components. Here, we study two organizations: *Jacobi* and *Gauss-Seidel*. The choice may have a crucial impact on both numerical stability and accuracy.

We introduce the notion of *macro time step* and *micro time step*. The macro time step determines the communication points: how long the components can run independently from each other without losing accuracy. The micro time step determines the discretization points of each component between two communication points. The latter is usually determined by accuracy, stability and thus the numerical method used.

For the purpose of explaining Jacobi and Gauss-Seidel organizations we introduce an abstract system composed of two components: component *1* and component *2*. Then we can define the system using a continuous representation as:

(5) $$\begin{array}{l} \frac{d}{dt}x_{1}=f_{1}(t,x_{1},x_{2})\\ \frac{d}{dt}x_{2}=f_{2}(t,x_{1},x_{2}), \end{array}$$

where *x*_1_, *x*_2_ are solution vectors of the respective component. Note that although the organization is discussed by considering only two components, the basic principles carry over the general case of *n* components.

#### 2.4.1. Jacobi organization

**Figure 1:**
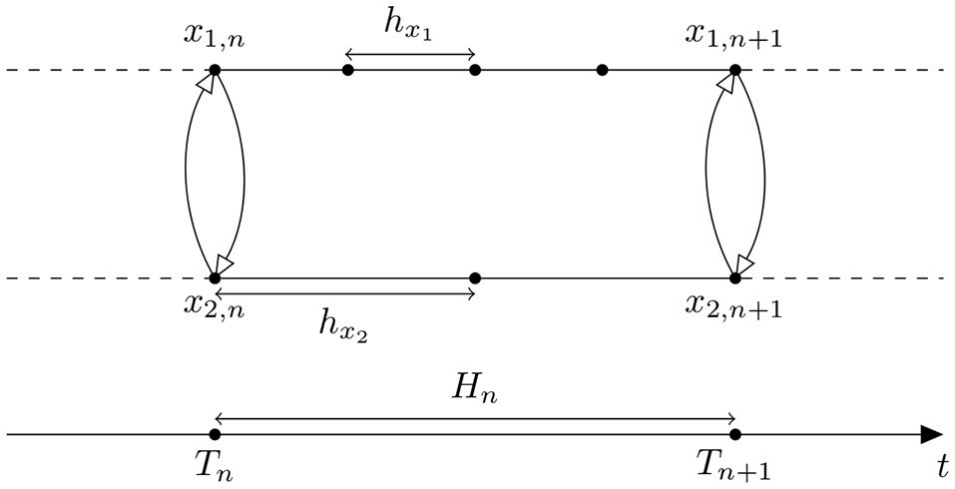
**Discretization in time of System (5) using Jacobi organization**. White triangle arrows correspond to the variables exchanged between component *1* and component *2*. *H*_*n*_ is a macro time step of System (5), *h*_*x*_1__, *h*_*x*_2__ are micro time steps of the component *1* and *2* respectively.

Jacobi organization in the system with two components leads to the interaction shown in Figure 1. In order to make a step from time *T*_*n*_ to *T*_*n*+1_ each component gets variables exchanged at time *T*_*n*_ (white triangle arrows on Figure 1). Then, the components are integrated to the time point *T*_*n*+1_.

In Jacobi organization System (5) can be rewritten in the form:

(6) $$\begin{array}{l} \frac{d}{dt}x_{1}=f_{1}(t,x_{1},{\tilde{x}}_{2,n+1})\\ \frac{d}{dt}x_{2}=f_{2}(t,{\tilde{x}}_{1,n+1},x_{2}), \end{array}$$

where ${\tilde{x}}_{1,n+1}$ and ${\tilde{x}}_{2,n+1}$ are approximations of the exchanged variables *x*_1_ and *x*_2_ at time *T*_*n*+1_, respectively. This organization works well in parallel computations since no component needs to wait for the other.

#### 2.4.2. Gauss-seidel organization

**Figure 2:**
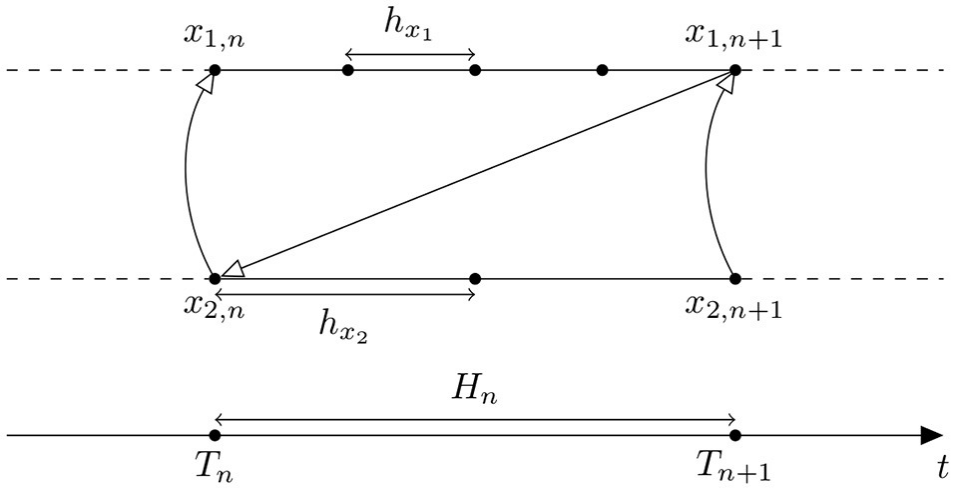
**Discretization in time of System (5) using Gauss-Seidel organization**. White triangle arrows correspond to the variables exchanged between the component *1* and the component *2*. *H*_*n*_ is a macro time step in System (5), *h*_*x*_1__, *h*_*x*_2__ are micro time steps of the component *1* and *2*, respectively.

The Gauss-Seidel organization updates each component in a sequential order (Figure 2). Let the component *1* be the leading component in System (5). Then, after the solution has been communicated at time *T*_*n*_ (white triangle arrow on Figure 2), the component *1* proceeds until *T*_*n*+1_. Then the calculated solution of the component *1* at time *T*_*n*+1_ can be used by the component *2*. This organization has been used by Mattioni and Le Novère (2013).

The Gauss-Seidel organization allows to eliminate the error introduced by one solution approximation in System (7).

(7) $$\begin{array}{l} \frac{d}{dt}x_{1}=f_{1}(t,x_{1},{\tilde{x}}_{2,n+1})\\ \frac{d}{dt}x_{2}=f_{2}(t,x_{1},x_{2}), \end{array}$$

where ${\tilde{x}}_{2,n+1}$ is an approximated solution of the component *2* as in Equation (6). However while Jacobi organization preserves parallelity of the integration, the Gauss-Seidel organization imposes a sequence of computation which reduces parallelity (Skelboe, 1992).

### 2.5. Approximation of exchanged variables

We aim to simulate two components with reciprocal data dependencies, that is the solution of each component depends on the solution of the other component at each integration time step. It can happen that the information is not available at a certain time point. For instance, the implicit methods require exchanged variable values and state variables from the current time point *T*_*n*+1_ as shown in System (6) and in System (7). Then an approximation of exchanged variables can be considered.

Here we will compare two approximation strategies, so called *Mode 1* and *Mode 3* introduced in Skelboe (2000). Mode 1 implies a constant extrapolation ${\tilde{x}}_{n+1}\equiv x_{n}$, so that the solution at the previous time step *n* is used when required. A second-order polynomial is applied in Mode 3. It uses the data from the previous time steps to approximate the value at time *T*_*n*+1_ Equation (8).

(8) $${\tilde{x}}_{n\;+\;1}=x_{n\;+\;1}^{p2}={\bar{\alpha }}_{1}x_{n}+{\bar{\alpha }}_{2}x_{n\;-\;1}+{\bar{\alpha }}_{3}x_{n\;-\;2}$$

$$\begin{array}{l} {\bar{\alpha }}_{1}=1-{\bar{\alpha }}_{2}-{\bar{\alpha }}_{3},{\bar{\alpha }}_{2}=\frac{\gamma_{n+1}(\gamma_{n+1}+\delta_{n+1})}{1-\delta_{n+1}},\\ \;{\bar{\alpha }}_{3}=\frac{\gamma_{n+1}(\gamma_{n+1}+1)}{\delta_{n+1}(\delta_{n+1}-1)}, \end{array}$$

where γ_*n*+1_ = *h*_*n*+1_/*h*_*n*_ and δ_*n*+1_ = 1 + *h*_*n*−1_/*h*_*n*_. In general, Equation (8) can be easily extended to a continuous extrapolation. In that case, the parameters ${\overset{-}{\alpha }}_{1},{\overset{-}{\alpha }}_{2},{\overset{-}{\alpha }}_{3}$ are functions of time. We use Equation (8) as a predictor in the estimate of the local discretization error and as an approximator of the exchanged variables in Mode 3.

### 2.6. Test case

**Figure 3:**
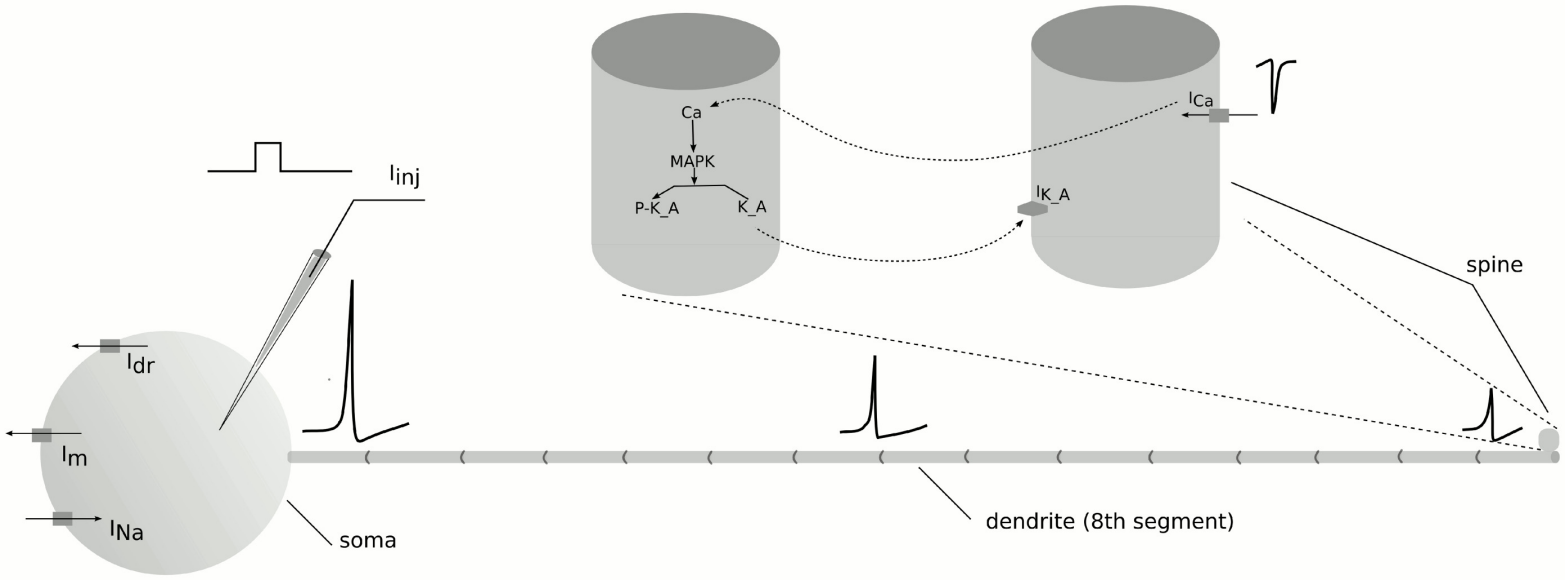
**A schematic representation of the multiscale neuron assembly**. A compartmental structure of the neuron is depicted together with the communication signals between electrical and biochemical models of the spine. The neuron consists of 17 compartments: a soma, 15 subcompartments of a dendrite and a spine. The morphological and physiological details are given in Supplementary Model Details.

In computational neuroscience both the electrical and the chemical signaling play a crucial role in the studies of learning and memory mechanisms. Synaptic input integration, signal propagation along the dendrites and action potential generation can often be modeled as electrical processes. These processes act on a timescale of about 0.001 s and a spatial scale from a few 100 μm to a few millimeters. The chemical processes typically span the timescales from a few seconds to months and years and can act within 1 μm. In our multiscale test system we span two levels of neural organization: cellular and subcellular levels. We model the electrical dynamics of a single neuron and biochemical processes in its *spine* (Figure 3).

Models of bistable chemical switches are commonly explored in the studies of long-term memory formation, in particular LTP and LTD. We choose the mitogen-activated protein kinase (MAPK) signaling model used in the study of homeostatic regulation of excitability at the scale of a single synapse by Bhalla (2011). The model exhibits bistability. It changes from the inactive to the active stable state by means of receptor phosphorylation caused by a calcium signal. To simulate the calcium influx we model a compartmentalized cell of a regular-spiking neuron (Pospischil et al., 2008). A current of 0.09·10^−9^ A is applied to the *soma* of the cell for 5 s. The change in the membrane potential activates voltage dependent sodium and potassium channels and a spike train propagates through the axial resistance to the spine. Spine depolarization activates voltage dependent calcium channels and a calcium current flows into the spine. Calcium influx triggers multiple signaling cascades on a sub-cellular level. *P-MAPK* becomes active, phosphorylates potassium channels *K_A* and leads to their non-conductivity. Then the biochemical model settles at its second steady state. The parameters of both models are given in Supplementary Model Details.

#### 2.6.1. Communication signals

We use Ca^2+^ influx as a key signal in our multiscale model to activate the MAPK cascade. The Ca^2+^ current in the electrical model (*I*_*Ca*_) is transformed to the calcium injection rate to the biochemical model (*k*_*inj*_) as shown in Equation (9).

(9) $$k_{inj}=\frac{N_{e}}{2\;\cdot \;N_{A}\;\cdot \;vol}\;\cdot \;I_{Ca}\;\left[\frac{M}{s}\right],$$

where *N*_*e*_ is the number of electrons in one Coulomb which roughly equals 6.242 · 10^18^, *N*_*A*_ is Avogadro's constant and *vol* is the volume of the spine compartment.

In turn, the biochemical model provides calcium concentration [*Ca*] and the fraction of active (non-phosphorylated) potassium channels in the spine $\frac{\left[K\text{\_}A\right]}{\left[K_{base}\right]}$. The fraction is used in the conductance evolution of the A-type potassium current in the electrical model Equation (10).

(10) $$g_{K_{A}}={\bar{g}}_{K_{A}}\frac{\left[K\_A\right]}{\left[K_{base}\right]}\;\left[S\right],$$

where ḡ_*K*_*A*__ is the maximum potassium conductance. Calcium concentration [*Ca*] is used in the Nernst potential recalculations for the calcium ion in the spine (see Supplementary Model Details).

#### 2.6.2. Mathematical formulation

The compartmental modeling with the HH formalism in the electrical component defines 17 subsequent electrical circuits or 24 ODEs (see Supplementary Model Details). Chemical reactions in the spine are defined by reaction-rate equations constituting a non-linear system of 18 ODEs (see Supplementary Model Details). Considering the communication signals System (5) can be reformulated in the following form:

(11) $$\begin{array}{l} \frac{d}{dt}x_{chem}=f_{chem}(t,x_{chem},g_{1}(x_{elec},x_{chem}))\\ \frac{d}{dt}x_{elec}=f_{elec}(t,g_{2}(x_{chem}),x_{elec}), \end{array}$$

where *g*_1_ and *g*_2_ are the output functions from the electrical and the biochemical component respectively.

(12) $$\begin{array}{l} g_{1}(x_{elec},x_{chem})=C_{1}(x_{elec,i}+C_{2}ln(x_{chem,j})-C_{3})x_{elec,k}^{2}x_{elec,l}\\ g_{2}(x_{chem})=\{\begin{array}{l} x_{chem,m}\\ C_{4}x_{chem,n} \end{array}, \end{array}$$

where *C*_1_..*C*_4_ are constants; the indices *i*..*m* correspond to positions of variables in the solution vector *x* at time *t*:

*i* - potential in the spine [V];

*j, m* - calcium concentration in the spine [M];

*k* - probability for an s gate being opened (calcium channel activation);

*l* - probability for an r gate being opened (calcium channel inactivation);

*n* - concentration of active (non-phosphorylated) potassium channels in the spine [M].

### 2.7. Implementation and simulation

The electrical and biochemical models, numerical disretization methods, Crank-Nicholson on a staggered grid, RK4 and BDF2, along with the adaptive step size controller was implemented in MATLAB®. The Crank-Nicholson on a staggered grid and the RK4 methods were used to solve the electrical and the biochemical component respectively. The adaptive step size controller was used only when both the electrical and the biochemical models were discretized with the BDF2 method.

**Figure 4:**
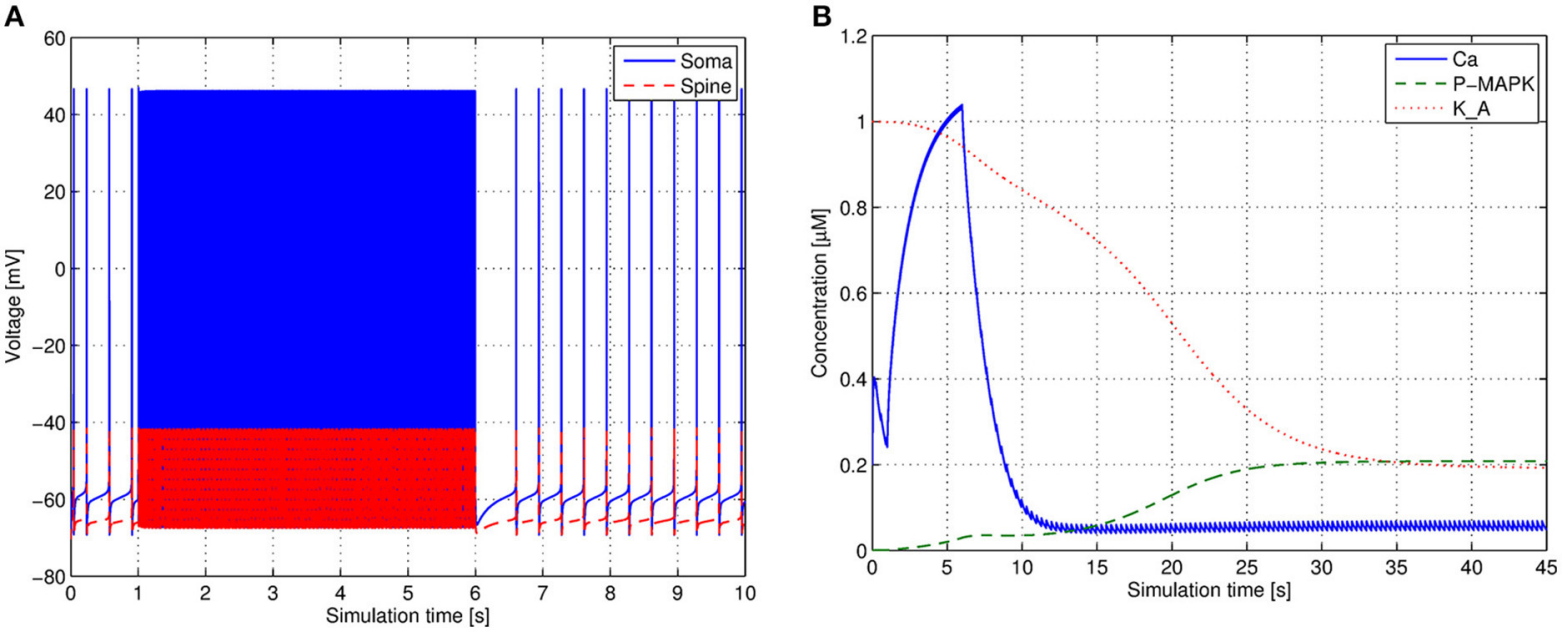
**System solution used as a reference for method evaluation**. **(A)** Voltage traces of soma and spine on the interval [0;10] s of simulation time. **(B)** Concentration traces of the characteristic molecules *Ca, P-MAPK* and *K_A* in the biochemical model.

Often a reference solution of the system is required for the numerical method evaluation. To obtain the reference solution we solve our system using *ode15s* MATLAB® function. *ode15s* is a solver designed for stiff problems. It is a quasi-constant step size implementation of the backward differentiation methods (Shampine and Reichelt, 1997). We set *RelTol* and *AbsTol* parameters to 10^−9^ and 10^−12^ respectively to achieve high accuracy in the reference solution. The Figures 4A,B depict the traces of a few most representative variables from the electrical and the biochemical components in the system respectively.

In our simulations in the proposed methods we used macro time step equal micro time step, that is *H*_*n*_ = *h*_*x*_1__ = *h*_*x*_2__. In general, the macro time step does not have to be equal the micro time step. For example, one can imagine to reduce the number of communication points when appropriate in order to optimize the data flow in parallel simulations. Another example is the multirate methods where each component can be solved with its own discretization time step, that is *h*_*x*_*i*__ ≠ *h*_*x*_*j*__. In this case, the macro time step is different from the micro time step at least for one of the components.

Two input parameters, *relTOL* and *absTOL*, are required by the error estimation step in the adaptive algorithm. The latter one, the absolute error tolerance *absTOL*, determines the accuracy when the solution approaches zero. In order to reduce the number of parameters we replaced *absTOL* in Equation (4) with the relative tolerance multiplied with a value representing a typical size of the corresponding solution component, *absTOL*_*i*_ = *relTOL* · |*Ytypical, i*|. We assigned *Ytypical* to the reference solution vector chosen at an arbitrary point of time during the stimulation.

### 2.8. Evaluation

We apply visual inspection to estimate the global error of the method by plotting the order-relative slopes, first- and second-order declines, in each figure in Section 3 (dashed lines). Visual inspection can also be used to understand whether an obtained solution deviates from the reference one. However in order to compare the accuracy of the methods a more rigorous technique is required.

A typical way to evaluate the accuracy of a numerical approximation method is to calculate the relative error of the solution at a characteristic point (often taken to be the final) of the simulation. In our system, the calcium communication signal has a crucial impact on the biochemical component. Thus we took a solution of the calcium concentration to calculate the error. To verify our observations we applied the same evaluation technique to the voltage solution in the spine.

We propose a component-wise relative error represented by Equation (13).

(13) $$\epsilon =\frac{1}{N}\sum_{i}^{N}\left\|{\vec{r}}_{i}\right\|\;\cdot \;100\;[\%]$$

where ${\vec{r}}_{i}=\left({\overset{ˇ}{p}}_{i}-p_{i}\right)⊘{\overset{ˇ}{p}}_{i}$, ${\overset{ˇ}{p}}_{i}=\left(t_{{\overset{ˇ}{p}}_{i}},v_{{\overset{ˇ}{p}}_{i}}\right)$ and *p*_*i*_ = (*t*_*p*_*i*__, *v*_*p*_*i*__) are characteristic points on the reference and approximate solutions respectively and ⊘ stands for an element-wise division of the vectors. Thus two components, the time *t* and the magnitude *v*, are considered in our formula. We motivate this choice with the thought in mind that both the strength and the time of the event are important in our simulations. In our solutions we defined maxima on an arbitrary chosen interval of the simulation as our characteristic points. We located two maxima values (*N* = 2) on the interval between 1 and 2 s of the simulation time to calculate relative error ϵ Equation (13).

To look at the efficiency of the coupling method we plotted the error vs. the total number of function calls of the right hand side of the system of ODEs during the simulation time *T* = 2 *s*. In the BDF2 implementation the total number of function calls includes the function calls used to compute the Jacobian matrix. For the RK4 method the total number of function calls per step equals four. In the CN on staggered grid implementation we estimated the work as one function call plus one Jacobian evaluation. The latter is comparable to one function call per step.

## 3. Results

### 3.1. The BDF2-BDF2 coupling outperforms RK4-CN on a fixed step size grid

We have a stiff electrical component that is usually solved with the CN method on a staggered grid in neuroscience. We applied the RK4 method to the biochemical system (Bhalla, 2011). Both methods are fixed step size methods. The BDF2 method can also be used on a fixed step size grid however this can be inefficient due to the multiple iterations per step required in implicit methods.

**Figure 5:**
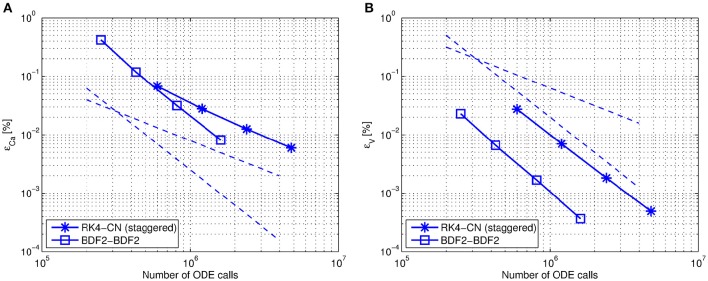
**Efficiency comparison between the RK4-CN (staggered) (“asterisk” markers) and the BDF2-BDF2 (“square” markers) coupling on a fixed step size grid**. The datapoints on each curve correspond to $h_{elec}=h_{chem}=\left\{2\cdot 10^{-5};1\cdot 10^{-5};5\cdot 10^{-6};2.5\cdot 10^{-6}\right\}$, respectively. The first discretization grid corresponds approximately to the one required by the fastest component when solved independently. The dashed lines correspond to the first- and second-order declines. The simulations were performed using Jacobi organization between the components with constant extrapolation of exchanged variables (Mode 1). **(A)** The relative error of the calcium solution versus the number of ODE calls. **(B)** The relative error of the voltage solution versus the number of ODE calls.

In Figure 5 we compare the accuracy of two different coupling methods, the BDF2-BDF2 coupling method and the CN on staggered grid with RK4 coupling on a fixed step size grid.

We observe that the accuracy of the decoupled BDF2 formula is preserved in the solutions. However an expected second-order accuracy of the calcium solution obtained with the RK4-CN coupling is lost (Figure 5A). This can happen due to insufficient accuracy of the exchanged variables. In the modified CN method calcium current is calculated at a half time step and then communicated at a whole time step. Thus the accuracy of the communicated variable is only of the first order. This can influence the order of the coupling method. We performed simulations using different order extrapolating techniques of the exchanged variables. However, we did not notice a sufficient order difference with respect to the calcium solution.

There is no visible accuracy loss in the voltage traces as shown in Figure 5B. We expect the order of a coupling method to be dependent on the strength of the coupling and thus vary for different systems. In our multiscale test problem we have a strong coupling influence of the electrical component on the biochemical and a weak coupling vice versa.

Overall the BDF2-BDF2 coupling shows more accurate results than the conventional methods on a given range of fixed step size grids. These promising outcomes indicate a further direction of the research.

### 3.2. The H211b controller leads to a smoother distribution of step sizes

An optimal behavior of the step size controller is when the step sizes that have to be taken do not have an extensive variation. Otherwise it increases the number of times the step size controller has to redo a step. A smooth distribution of step sizes leads to good performance of the integrator.

**Figure 6:**
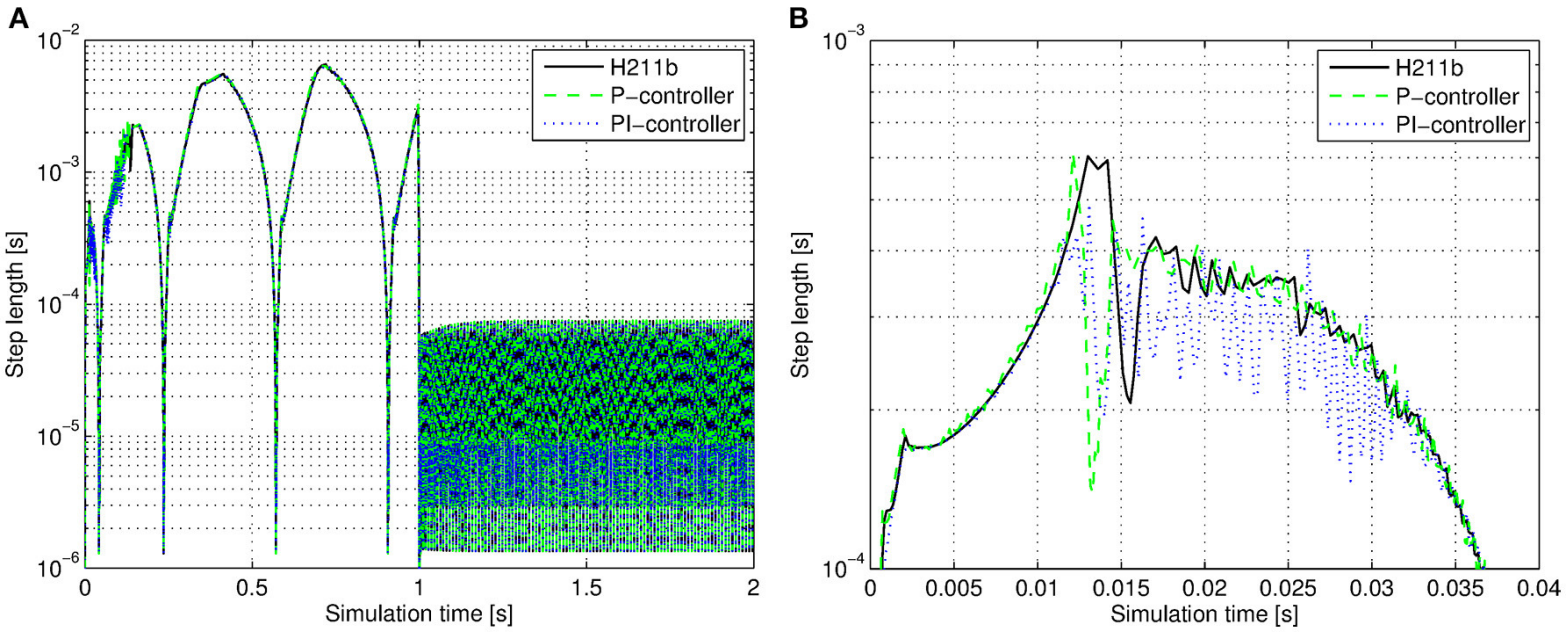
**The size of a step taken by the P-, PI-, H211b controllers as a function of simulation time evaluation. (A)** The time span of 2 s. After 1 s of simulation time a current stimulus is given to the soma that leads to a large increase in the firing rate. Hence the required time step becomes small. **(B)** Close up on time interval (0;0.04) s.

In Figure 6 we compare the P-, PI- and H211b controllers. The H211b controller shows a smoother step size variation at the beginning of the simulation. Thus we chose the H211b controller for our further observations.

### 3.3. An appropriate approximation of exchanged variables

**Figure 7:**
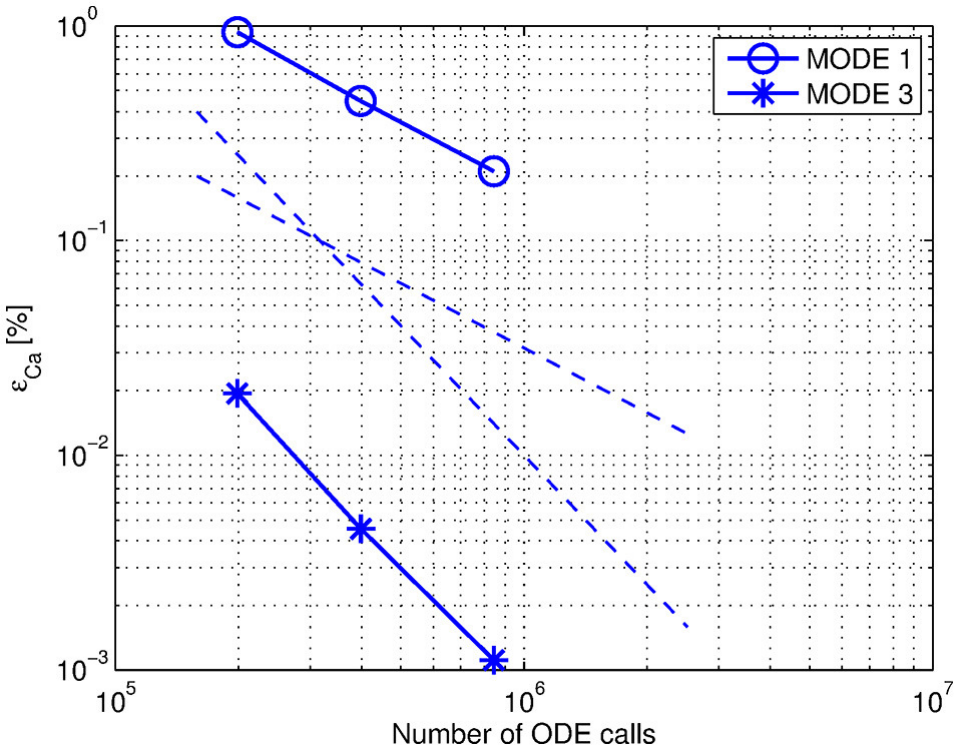
**Efficiency comparison between Mode 1 and Mode 3**. The simulations were performed using Jacobi organization in the system. The datapoints correspond to *relTOL* = {10^−5^; 10^−6^; 10^−7^}. The dashed lines are the first- and second-order declines.

In these simulations we compare the constant extrapolation (Mode 1) with the second-order polynomial extrapolation of exchanged variables (Mode 3) described in Section 2.5. We used Jacobi organization of the components and an adaptive step size controller with the BDF2 approximation method in these simulations. The relative errors both of calcium concentration ϵ_*Ca*_ and of voltage in the spine ϵ_*V*_ were calculated. An expected asymptotic behavior is observed in Mode 3 and only a first-order of coupling in Mode 1 (Figure 7). Accuracy measurements of the voltage solution do not show significant difference between the modes (not shown).

A significant advantage of the second-order polynomial extrapolation (Mode 3) over the constant extrapolation (Mode 1) with respect to solution accuracy demonstrates the importance of an appropriate choice between different approximation strategies of exchanged variables.

In general, approximated values can be of 0th order (Mode 1) if the coupling between the components is weak or *x*_*i*_(*t*) is slowly varying, otherwise the results will be inaccurate (Sand and Skelboe, 1992). Our results are perfectly in line with this observation since our test case has a strong coupling between the electrical and the biochemical component.

### 3.4. Organization of system components

**Figure 8:**
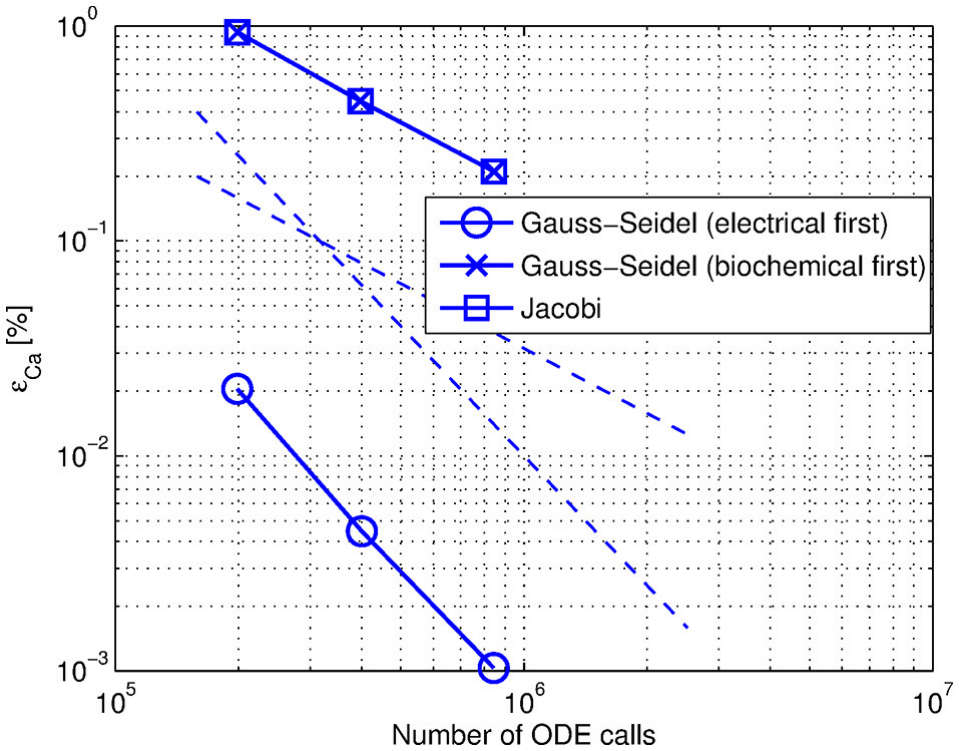
**Efficiency comparison between Jacobi, Gauss-Seidel with electrical component solved first and Gauss-Seidel with biochemical component solved first organizations**. The datapoints on the figure correspond to *relTOL* = {10^−5^; 10^−6^; 10^−7^}. The dashed lines are the first and second-order declines.

We investigate whether different organizations between the components in our system have an impact on the solution accuracy. Having a strong influence of the electrical component on the biochemical during the stimulation interval we predicted that by letting the electrical component lead the integration we could possibly avoid an approximation error of the exchanged variables and improve overall performance. The results we observe are consistent with our expectations (Figure 8). Gauss-Seidel organization with the electrical component solved first in Mode 1 lead to more accurate results.

We also compared different organizations in Mode 3. We did not notice any superiority of Gauss-Seidel organization with the electrical component solved first.

We conclude that Jacobi organization with the second-order polynomial approximation of the exchanged variables (Mode 3) can have similar accuracy as Gauss-Seidel organization with an appropriate ordering of system components in Mode 1.

## 4. Discussion

Several strategies have been proposed for coupling multiple components of a multiscale system on a behavioral level recently (Bhalla, 2011; Mattioni and Le Novère, 2013). One of the questions that has not been addressed in the research literature is mathematical justification of the coupling strategy. In this paper we discussed the problems of inefficiency and possible numerical instability that may arise while coupling multiple components comprising a multiscale system.

We introduced an implicit approximation method, two-step Backward Differentiation formula (BDF2) as a possible alternative to the conventional discretization methods used in neuroscience. This numerical method appealed to our interest for several reasons. First, it has been previously introduced as a decoupled implicit BDF2 formula by Skelboe (2000). Its stability properties and error propagation estimates within a coupled integration have been discussed. Second, the proposed error estimation allowed us to use the adaptive time-stepping algorithm described in Section 2.3.

In this paper we investigated the influence of such factors as an approximation of exchanged variables and different organizations of system components. We compared constant (Mode 1) and second-order polynomial (Mode 3) extrapolation of exchanged variables in our simulations. We also introduced two types of organization between the components, Jacobi and Gauss-Seidel. Our results show that an appropriate approximation of exchanged variables and organization of system components plays a significant role in efficient integration of the coupled system. Moreover, an application of the proposed integration method with the Jacobi-type organization of system components is well suited for parallel computations.

**Figure 9:**
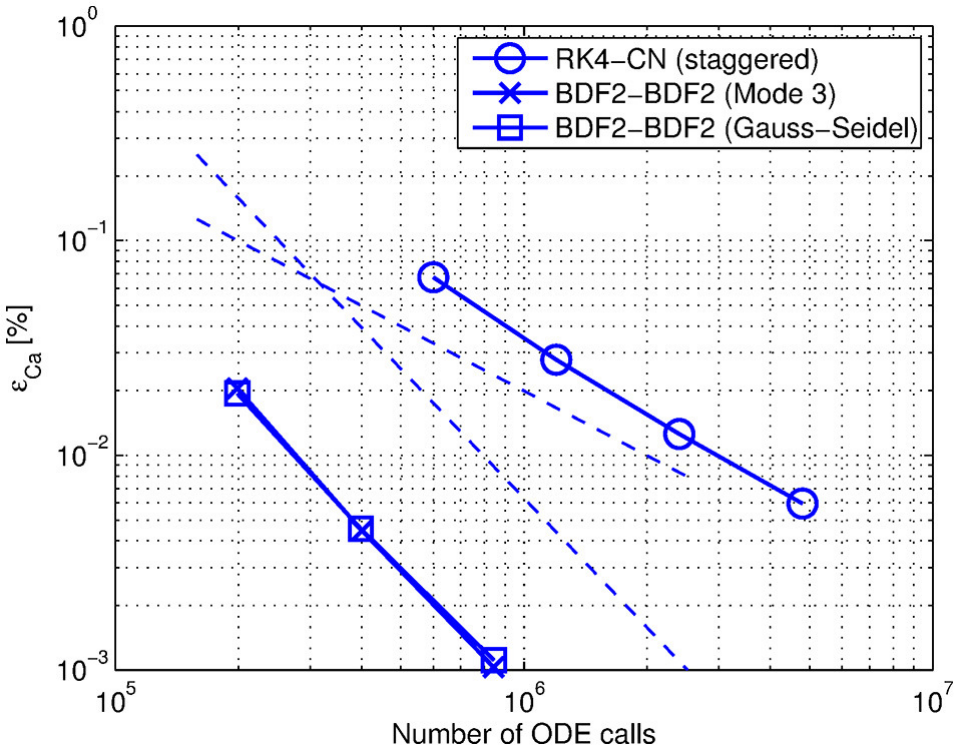
**Efficiency comparison between the proposed coupling method BDf2-BDF2 (“cross” and “square” markers) and the conventional coupling method RK4-CN (“circle” markers)**. The datapoints represent the error estimates of the solution obtained with *relTOL* = {10^−5^; 10^−6^; 10^−7^} and on the fixed grid with the step sizes $h_{elec}=h_{chem}=\left\{2\cdot 10^{-5};1\cdot 10^{-5};5\cdot 10^{-6};2.5\cdot 10^{-6}\right\}$ accordingly. The dashed lines correspond to the first and second-order declines.

The power and applicability of the method was demonstrated solving a multiscale test case that was designed as a prototype of models used in the scope of interest. In Figure 9 we combine the efficiency measurements of the solutions obtained with the adaptive step size BDF2-BDF2 coupling method and those obtained with the fixed step size coupling RK4 - CN (staggered) method. The BDF2-BDF2 coupling in Mode 3 with Jacobi organization (the curve with “square” markers) is comparable to the BDF2-BDF2 coupling in Mode 1 with Gauss-Seidel organization where the electrical component was solved first (the curve with “asterisk” markers). The proposed integration method allowed us to solve the system in a more efficient way. An application of the method and conclusions can be generalized for similar problems formulated by systems of ODEs and DAEs.

Multiscale problems usually span multiple time scales. The step sizes required for numerical stability and desired accuracy normally differ for different system components when solved separately. In this paper, we used macro time-stepping equal micro time-stepping for each component in order to address the questions of stability and accuracy of a coupled integration. Intuitively one would imagine to use small steps for the fastest changing components and larger steps in slow components in the hope of reducing the amount of computational and communication work, a so-called *multirate method*. To the knowledge of the authors an application of the multirate integration to the physical systems is a non trivial task with a limited choice of available methods. Skelboe (2000) mentions an applicability of the decoupled integration formula in a multirate mode with a waveform relaxation method. In Günther and Rentrop (1993) tested the multirate Rosenbrock-Wanner schemes on a highly integrated electric circuits. The method showed a potential for a computational speedup. Therefore, an extension of the proposed method to allow multirate integration can be further considered.

## Author contributions

All authors listed, have made substantial, direct and intellectual contribution to the work, and approved it for publication. MH, UB, EB, MD, and JH conceived, designed the experiments. EB and MH performed the experiments. MH, EB, UB, MD, and JH analyzed the results. EB wrote the paper.

## Funding

The research leading to these results has received funding from the European Union Seventh Framework Programme (FP7/2007-2013) under grant agreement no 604102 (HBP), the Swedish Research Council (Vetenskapsrådet), NIAAA (grant 2R01AA016022), Swedish e-Science Research Center, and an Erasmus Mundus Joint Doctoral program EuroSPIN.

### Conflict of interest statement

The authors declare that the research was conducted in the absence of any commercial or financial relationships that could be construed as a potential conflict of interest.
