# Supplementary material for: Efficient Integration of Coupled Electrical-Chemical Systems in Multiscale Neuronal Simulations
Source: Front Comput Neurosci. 2016 Sep 12;10:97. doi: 10.3389/fncom.2016.00097 (PMC5018489; doi:10.3389/fncom.2016.00097)
Supplement: Supplementary file 1 [file DataSheet1.PDF]

Numerical Methods

We focus on a class of numerical methods designed to solve ODEs with a given initial value (initial value problem (IVP)). Given an IVP in the form:

$$y' = f(t, y), \qquad y(t_0) = y_0, \tag{1}$$

the easiest way to approximate a solution is to use the Forward Euler method:

$$y_{n+1} = y_n + hf(t_n, y_n) \tag{2}$$

Here, y_n is the approximated solution at time point t_n; h is the size of the time step. The Forward Euler method is a first order numerical method, which means that the global error is proportional to the step size of discretization h.

The Crank-Nicholson method on a staggered grid

Michael Hines observed that the system of ordinary differential equations describing certain electrical phenomena has a very special structure which can be used in order to speed up the simulation [1]. His first observation was that the system is a stiff system such that implicit methods must be used in order to avoid very small stepsizes. Moreover, the ode system is highly nonlinear which would create the need for a Newton iteration in every integration step. However, some variables of the systems appear only linearly such that, at least for these variables, the Newton iteration provides the exact solution after one step. In [1] the method is described with a very special model in mind. In the present section, we will investigate the method in a more general setting.

Since the convergence properties have never been rigorously proven to the best knowledge of the authors we will do this in a first step. Further investigations concern its asymptotic properties.

Consider the following ode system,

$$\begin{aligned}\dot{x} &= a(y)x + b(y),\\ \dot{y} &= c(x) + d(x)y,\end{aligned}$$

where $x : [0, T] \longrightarrow \mathbb{R}^m$, $y : [0, T] \longrightarrow \mathbb{R}^n$ subject to the boundary conditions

$$x(0) = x_0, \quad y(0) = y_0.$$

Discretization. The numerical method on an equidistant grid with stepsize h can be written down as

$$\begin{aligned}x_{i+1} &= x_i + h\left[a(y_{i+1/2})\frac{x_{i+1} + x_i}{2} + b(y_{i+1/2})\right],\\ y_{i+3/2} &= y_{i+1/2} + h\left[c(x_{i+1}) + d(x_{i+1})\frac{y_{i+3/2} + y_{i+1/2}}{2}\right].\end{aligned}$$

In [1] Hines motivated the formula by the trapezoidal rule. In fact, it resembles rather the implicit midpoint rule. By using the staggered form of the method it becomes linearly implicit thus reducing the computational amount considerably. This is done at the cost of an additional intial value needed $y_{1/2}$ and the assumption of constant stepsizes. By Taylor expansion it is easily shown that the method has second order of accuracy. Therefore, it is appropriate to use a second order consistent approximation for $y(h/2)$. For the convergence considerations it is sufficient to consider the exact value $y_{1/2} = y(h/2)$ to be known.

In order to show the convergence of the method, 0-stability must be shown. The stability notion adapted to the method will be modelled following the ideas presented by Ascher and Petzold in [4]. Let for given sequences $\{x_i\}_{i=0}^{N(h)} \subset \Omega_x$ and $\{y_{i+1/2}\}_{i=0}^{N(h)} \subset \Omega_y$ be defined

$$\begin{aligned}\mathcal{N}_x(x,y)_i &= \frac{x_{i+1}-x_i}{h} - a(y_{i+1/2})\frac{x_{i+1}+x_i}{2} - b(y_{i+1/2}),\\ \mathcal{N}_y(x,y)_i &= \frac{y_{i+3/2}-y_{i+1/2}}{h} - c(x_{i+1}) - d(x_{i+1})\frac{y_{i+3/2}+y_{i+1/2}}{2}.\end{aligned}$$

Here, $N(h) = T/h$.

Definition 1. The method is 0-stable on $[0,T]$, if there exists $h_0 > 0$ and $K \in \mathbb{R}$ such that for any two sequences $\left\{x_i^j\right\}_{i=0}^{N(h)}$, $\left\{y_{i+1/2}^j\right\}_{i=0}^{N(h)}$, $j = 1,2$ it holds

$$\begin{aligned}&\max_{1\le i\le N(h)} \left\{|x_i^1 - x_i^2| + |y_{i+1/2}^1 - y_{1+1/2}^2|\right\}\\ &\le K \Big\{|x_0^1 - x_0^2| + |y_{1/2}^1 - y_{1/2}^2|\\ &+ \max_{1\le i\le N(h)} |\mathcal{N}_x(x^1,y^1)_i - \mathcal{N}_x(x^2,y^2)| + \max_{1\le i\le N(h)} |\mathcal{N}_y(x^1,y^1)_i - \mathcal{N}_y(x^2,y^2)|\Big\}\end{aligned}$$

In order to be specific let the functions a, b, c, d be defined on certain bounded domains $\Omega_x \subseteq \mathbb{R}^m$, $\Omega_y \subseteq \mathbb{R}^n$ with $x_0 \in \Omega_x$ and $y \in \Omega_y$. We assume the following conditions to be fulfilled:

1. $a : \Omega_y \longrightarrow \mathbb{R}^{m\times m}$, $b : \Omega_y \longrightarrow \mathbb{R}^m$, $c : \Omega_x \longrightarrow \mathbb{R}^n$, $d : \Omega_x \longrightarrow \mathbb{R}^{n\times n}$.
2. a, b, c, d are Lipschitz continuous on their respective domains with the Lipschitz constants L_a, L_b, L_c, L_d respectively.
3. Since Ω_x and Ω_y are bounded, Lipscitz continuity implies boundedness of a, b, c, d. Let the corresponding bounds be denoted by M_a, M_b, M_c, M_d.

Under these conditions the following theorem is not hard to prove.

Theorem 2. *Under the given assumptions, the initial value problem has a unique solution which can be extended to the boundary of $[0,T] \times \Omega_x \times \Omega_y$.*

Theorem 3. *Hines' method is (zero-)stable.*

Proof. Let $\left\{x_i^j\right\}_{i=0}^{N(h)} \subset \Omega_x$ and $\left\{y_{i+1/2}^j\right\}_{i=0}^{N(h)} \subset \Omega_y$ be given as in the definition. Let $\alpha_i^j = \mathcal{N}_x(x^j,y^j)_i$ and $\beta_i^j = \mathcal{N}_y(x^j,y^j)_i$. Let $h_0 = \min\left\{M_a^{-1}, M_d^{-1}\right\}$. In that case, one obtains immediately the estimates

$$\begin{aligned}\left|(I - \frac{h}{2}a(y))^{-1}\right| \le 2, \quad \left|I - \frac{h}{2}a(y)\right| \le 2 \text{ for all } y \in \Omega_y \text{ and all } h \le h_0;\\ \left|(I - \frac{h}{2}d(x))^{-1}\right| \le 2, \quad \left|I - \frac{h}{2}d(x)\right| \le 2 \text{ for all } x \in \Omega_x \text{ and all } h \le h_0.\end{aligned}$$

Moreover, we have

$$
\begin{aligned}
&\left|(I-\frac{h}{2}a(y^1_{y+1/2}))^{-1}-(I-\frac{h}{2}a(y^2_{y+1/2}))^{-1}\right| \\
&\leq \left|(I-\frac{h}{2}a(y^1_{y+1/2}))^{-1}((I-\frac{h}{2}a(y^2_{y+1/2}))-(I-\frac{h}{2}a(y^1_{y+1/2})))(I-\frac{h}{2}a(y^2_{y+1/2}))^{-1}\right| \\
&\leq \left|(I-\frac{h}{2}a(y^1_{y+1/2}))^{-1}\right|\frac{h}{2}\left|a(y^1_{y+1/2})-a(y^2_{y+1/2})\right|\left|(I-\frac{h}{2}a(y^2_{y+1/2}))^{-1}\right| \\
&\leq 2\frac{h}{2}L_a\left|y^1_{y+1/2}-y^2_{y+1/2}\right|2 \\
&\leq Ch\left|y^1_{y+1/2}-y^2_{y+1/2}\right|.
\end{aligned}
$$

Here and in the following, C denotes a generic constant independend of the sequences $\left\{x^j_i\right\}_{i=0}^{N(h)}$ and $\left\{y^j_{i+1/2}\right\}_{i=0}^{N(h)}$ and the stepsize h. A further useful estimate is the following one,

$$
\begin{aligned}
&\left|(I-\frac{h}{2}a(y))^{-1}(I+\frac{h}{2}a(y))-I\right| \\
&\leq \left|(I-\frac{h}{2}a(y))^{-1}\right|\left|(I+\frac{h}{2}a(y))-(I-\frac{h}{2}a(y))\right| \\
&\leq Ch.
\end{aligned}
$$

Hence,

$$
\left|(I-\frac{h}{2}a(y))^{-1}(I+\frac{h}{2}a(y))\right| \leq 1+Ch.
$$

Since the sequences $\left\{x^j_i\right\}_{i=0}^{N(h)} \subset \Omega_x$ and $\left\{y^j_{i+1/2}\right\}_{i=0}^{N(h)} \subset \Omega_y$ are bounded, the definition of α_i and β_i leads to the estimates

$$
\alpha_i \leq C/h, \quad \beta_i \leq C/h.
$$

Using the definition of α_i, we obtain

$$
\begin{aligned}
\left|x^1_{i+1}-x^2_{i+1}\right| \leq \Big|&(I-\frac{h}{2}a(y^1_{i+1/2}))^{-1}(I+\frac{h}{2}a(y^1_{i+1/2}))x^1_i+h(I-\frac{h}{2}a(y^1_{i+1/2}))^{-1}(b(y^1_{i+1/2})+\alpha^1_i) \\
&-(I-\frac{h}{2}a(y^2_{i+1/2}))^{-1}(I+\frac{h}{2}a(y^2_{i+1/2}))x^2_i+h(I-\frac{h}{2}a(y^2_{i+1/2}))^{-1}(b(y^2_{i+1/2})+\alpha^2_i)\Big|.
\end{aligned}
$$

The terms will be estimated individually.

$$
\begin{aligned}
&\left|(I-\frac{h}{2}a(y^1_{i+1/2}))^{-1}(I+\frac{h}{2}a(y^1_{i+1/2}))x^1_i-(I-\frac{h}{2}a(y^2_{i+1/2}))^{-1}(I+\frac{h}{2}a(y^2_{i+1/2}))x^2_i\right|\\
\leq\quad & \left|(I-\frac{h}{2}a(y^1_{i+1/2}))^{-1}(I+\frac{h}{2}a(y^1_{i+1/2}))x^1_i-(I-\frac{h}{2}a(y^2_{i+1/2}))^{-1}(I+\frac{h}{2}a(y^1_{i+1/2}))x^1_i\right|\\
&+\left|(I-\frac{h}{2}a(y^2_{i+1/2}))^{-1}(I+\frac{h}{2}a(y^1_{i+1/2}))x^1_i-(I-\frac{h}{2}a(y^2_{i+1/2}))^{-1}(I+\frac{h}{2}a(y^2_{i+1/2}))x^1_i\right|\\
&+\left|(I-\frac{h}{2}a(y^2_{i+1/2}))^{-1}(I+\frac{h}{2}a(y^2_{i+1/2}))x^1_i-(I-\frac{h}{2}a(y^2_{i+1/2}))^{-1}(I+\frac{h}{2}a(y^2_{i+1/2}))x^2_i\right|\\
\leq\quad & \left|(I-\frac{h}{2}a(y^1_{i+1/2}))^{-1}-(I-\frac{h}{2}a(y^2_{i+1/2}))^{-1}\right|\left|(I+\frac{h}{2}a(y^1_{i+1/2}))x^1_i\right|\\
&+\left|(I-\frac{h}{2}a(y^2_{i+1/2}))^{-1}\right|\frac{h}{2}\left|a(y^1_{i+1/2})-a(y^2_{i+1/2})\right|\left|x^1_i\right|\\
&+\left|(I-\frac{h}{2}a(y^2_{i+1/2}))^{-1}(I+\frac{h}{2}a(y^2_{i+1/2}))\right|\left|x^1_i-x^2_i\right|\\
\leq\quad & Ch\left|y^1_{y+1/2}-y^2_{y+1/2}\right|2R_x\\
&+2L_a\frac{h}{2}\left|y^1_{y+1/2}-y^2_{y+1/2}\right|R_x\\
&+(1+Ch)\left|x^1_i-x^2_i\right|\\
\leq\quad & (1+Ch)\left|x^1_i-x^2_i\right|+Ch\left|y^1_{y+1/2}-y^2_{y+1/2}\right|.
\end{aligned}
$$

The next term allows for the following estimates,

$$
\begin{aligned}
&\left|(I-\frac{h}{2}a(y^1_{i+1/2}))^{-1}(b(y^1_{i+1/2})+\alpha^1_i)-(I-\frac{h}{2}a(y^2_{i+1/2}))^{-1}(b(y^2_{i+1/2})+\alpha^2_i)\right|\\
\leq\quad & \left|(I-\frac{h}{2}a(y^1_{i+1/2}))^{-1}(b(y^1_{i+1/2})+\alpha^1_i)-(I-\frac{h}{2}a(y^2_{i+1/2}))^{-1}(b(y^1_{i+1/2})+\alpha^1_i)\right|\\
&+\left|(I-\frac{h}{2}a(y^2_{i+1/2}))^{-1}(b(y^1_{i+1/2})+\alpha^1_i)-(I-\frac{h}{2}a(y^2_{i+1/2}))^{-1}(b(y^2_{i+1/2})+\alpha^2_i)\right|\\
\leq\quad & Ch\left|y^1_{y+1/2}-y^2_{y+1/2}\right|\left|b(y^1_{i+1/2})+\alpha^1_i\right|\\
&+2\left(L_b\left|y^1_{y+1/2}-y^2_{y+1/2}\right|+\left|\alpha^1_i-\alpha^2_i\right|\right)\\
\leq\quad & C\left|y^1_{y+1/2}-y^2_{y+1/2}\right|+C\left|\alpha^1_i-\alpha^2_i\right|
\end{aligned}
$$

With the latter two estimates, we obtain,

$$
\left|x^1_{i+1}-x^2_{i+1}\right|\leq(1+Ch)\left|x^1_i-x^2_i\right|+Ch\left|y^1_{y+1/2}-y^2_{y+1/2}\right|+Ch\left|\alpha^1_i-\alpha^2_i\right|.
$$

A similar estimate leads to

$$
\begin{aligned}
\left|y^1_{i+3/2}-y^2_{i+3/2}\right| &\leq(1+Ch)\left|y^1_{i+1/2}-y^2_{i+1/2}\right|+Ch\left|x^1_{i+1}-x^2_{i+1}\right|+Ch\left|\beta^1_i-\beta^2_i\right|\\
&\leq(1+Ch)\left|y^1_{i+1/2}-y^2_{i+1/2}\right|+Ch\left|x^1_i-x^2_i\right|+Ch\left|\beta^1_i-\beta^2_i\right|+Ch^2\left|\alpha^1_i-\alpha^2_i\right|.
\end{aligned}
$$

Set

$$
z^j_i=\begin{pmatrix}x^j_i\\ y^j_{i+1/2}\end{pmatrix},\quad \delta^j_i=\begin{pmatrix}\alpha^j_i\\ \beta^j_i\end{pmatrix}.
$$

With this definitions, we have the estimate

$$\left|z_{i+1}^1 - z_{i+1}^2\right| \le (1+Ch)\left|z_i^1 - z_i^2\right| + Ch\left|\delta_i^1 - \delta_i^2\right|.$$

A standard estimate, see e.g. [4] p.42, yields the desired inequality,

$$\left|z_i^1 - z_i^2\right| \le K\left\{\left|z_0^1 - z_0^2\right| + \max_{1\le i\le N(h)}\left|\delta_i^1 - \delta_i^2\right|\right\}.$$

□

By a standard Taylor expansion it is easily verified that, for the local trucncation errors,

$$\tau_{x,i} := \mathcal{N}_x(x(\cdot), y(\cdot))_i, \quad \tau_{y,i} := \mathcal{N}_y(x(\cdot), y(\cdot))_i$$

it holds

$$\tau_{x,i} = O(h^2), \quad \tau_{y,i} = O(h^2).$$

Using the stability estimate, we see immediately that Hines' method is second order accurate.

Absolute stability of Hines' method. Hines' method belongs to the class of partioned discretization methods. The classical theory of A-stability of numerical methods for ordinary differential equations relies on the scalar test equation

$$z' = \lambda z, \quad \lambda \in \mathbb{C}.$$

In the context of partitioned methods this test equation is not appropriate. We will need a test system which consists of at most two scalar equations. To the best knowledge of the authors there is no widely accepted test equation available. In the following considerations we will follow the proposal by Strehmel and Weiner in [2]:

$$\begin{aligned}\dot{x} &= \mu x + ay,\\ \dot{y} &= bx + \lambda y.\end{aligned} \tag{3}$$

The coefficients should be chosen in such a way that they reflect the properties of the intended application:

1. The system as a whole should be asymptotically stable. This is the case if and only if $ab < \mu\lambda$ holds.
2. The individual equation should be asymptotically stable if the other component is held fixed. This is the case if and only if $\mu, \lambda < 0$.

Let these conditions be fulfilled in the following.

The application of Hines' method to the test system (3) leads to a linear recursion

$$\begin{pmatrix} x_{i+1} \\ y_{i+3/2} \end{pmatrix} = C \begin{pmatrix} x_i \\ y_{i+1/2} \end{pmatrix}$$

where

$$C = \begin{pmatrix} \alpha & \frac{ha}{1-h\mu/2} \\ \alpha\frac{hb}{1-h\lambda/2} & \frac{h^2ab}{(1-h\mu/2)(1-h\lambda/2)} \end{pmatrix}, \quad \alpha = \frac{1+h\mu/2}{1-h\mu/2}, \quad \beta = \frac{1+h\lambda/2}{1-h\lambda/2}. \tag{4}$$

From our assumptions we have $|\alpha|, |\beta| < 1$. The recursion is asymptotically stable if the spectral radius of C is less than one. The characteristic polynomial becomes

$$\varphi(\nu) = \nu^2 - \big(\alpha + \beta + \frac{h^2ab}{(1-h\mu/2)(1-h\lambda/2)}\big).$$

A general discussion of the spectral radius is hard. However, in two special cases, conclusions can easily be drawn:

- Assume $ab = 0$. In this case, the equations are partly decoupled. The eigenvalues become $\nu_1 = \alpha$ and $\nu_2 = \beta$. So the discretization is asymptotically stable for all parameters.
- If $ab < 0$, there exist always a stepsize h such that the spectral radius is larger than one.

The second case is particularly interesting. It is well-known the the implicit midpoint rule is A-stable. If it will be applied to the system (3) as a whole (i.e. not in the partitioned form), the discrete system will be stable independent of the stepsize. However, if applied in the form of Hines' method, the stability domain becomes bounded.

The Classical Runge-Kutta method

In 1901, Kutta applied the midpoint rule and the explicit Euler approximation to the original problem in Eq. 1 and thus introduced the first instance in a class of numerical methods.

One of the Runge-Kutta methods has been more popular than the others in neuroscience. It is the fourth order Runge-Kutta method shown in Eq. 5, and is often referred to as The Classical Runge-Kutta method.

$$y_{n+1} \approx y_n + \frac{h}{6}\left(k_1 + 2k_2 + 2k_3 + k_4\right) \tag{5}$$

where

$$\begin{aligned} k_1 &= f(t_n, y_n) \\ k_2 &= f\left(t_n + \frac{h}{2}, y_n + \frac{h}{2}k_1\right) \\ k_3 &= f\left(t_n + \frac{h}{2}, y_n + \frac{h}{2}k_2\right) \\ k_4 &= f\left(t_n + h, y_n + hk_3\right) \end{aligned} \tag{6}$$

The Classical Runge-Kutta method is an explicit numerical method known to have a bounded stability region. For a stiff problem this causes a solution to become unstable if a small enough discretization time step has not been chosen.

Backward Differentiation Formula methods

Eq. 1 can be solved either using integral approach or using methods based on differentiation, so called the Backward differentiation Formula (BDF) methods. These methods were introduced by Curtiss and Hirschfelder in [3].

The Backward differentiation formulas with an order less than three are often used for stiff systems due to their property of being A-stable. We are interested in the second-order backward differentiation formula (BDF2). On an equidistant grid the BDF2 formula has the form:

$$\frac{2}{3}y_{n+1} - 2y_n + \frac{1}{2}y_{n-1} = hf_{n+1}(t_{n+1}, y_{n+1}) \tag{7}$$

On a non-uniform grid the BDF2 method can be formulated as:

$$y_{n+1} = \alpha_1 y_n + \alpha_2 y_{n-1} + \beta h_{n+1} f(t_{n+1}, y_{n+1}) \tag{8}$$

where

$$\gamma_{n+1} = h_{n+1}/h_n \tag{9}$$

$$\alpha_1 = 1 - \alpha_2 \tag{10}$$

$$\alpha_2 = -\gamma_{n+1}^2/(2\gamma_{n+1} + 1) \tag{11}$$

$$\beta = (\gamma_{n+1} + 1)/(2\gamma_{n+1} + 1) \tag{12}$$

References

1. Hines M (1984) Efficient computation of branched nerve equations. International journal of bio-medical computing 15: 69–76.

2. Strehmel K, Weiner R (1984) Partitioned adaptive Runge-Kutta methods and their stability. Numerische Mathematik 45: 283–300.

3. Curtiss C, Hirschfelder J (1952) Integration of stiff equations. Proceedings of the National Academy of Sciences of the United States of America 38: 235.

4. Ascher UM, Petzold LR (1998) Computer methods for ordinary differential equations and differential-algebraic equations, volume 61. Siam.
