# Supplementary material for: Efficient Integration of Coupled Electrical-Chemical Systems in Multiscale Neuronal Simulations
Source: Front Comput Neurosci. 2016 Sep 12;10:97. doi: 10.3389/fncom.2016.00097 (PMC5018489; doi:10.3389/fncom.2016.00097)
Supplement: Supplementary file 2 [file DataSheet2.PDF]

Instability Example in the Coupled Simulation

What can be the consequences of a coupled simulation when multiple numerical solvers are run simultaneously and exchange state variables at some communication points?

Let's take a test equation proposed by Strehmel and Weiner in [1]:

$$\begin{aligned} x' &= \mu x + ay \\ y' &= bx + \lambda y \end{aligned} \tag{1}$$

where $\mu, \lambda < 0$ and $ab < \mu\lambda$. Under these conditions, the individual equations are asymptotically stable as is the system.

The backward Euler numerical approximation method applied to the system (1) will lead to the following form:

$$\begin{aligned} x_{n+1} &= x_n + h(\mu x_{n+1} + ay_{n+1}) \\ y_{n+1} &= y_n + h(bx_{n+1} + \lambda y_{n+1}) \end{aligned} \tag{2}$$

where h is the discretization time step.

In a coupled simulation when the components X and Y are solved simultaneously and separately the variables have to be exchanged each communication time step H as shown in (3).

$$\begin{aligned} x_{n+1} &= x_n + H(\mu x_{n+1} + a\mathbf{y_n}) \\ y_{n+1} &= y_n + H(b\mathbf{x_n} + \lambda y_{n+1}) \end{aligned} \tag{3}$$

where H is the *macro time step* that determines the communication points between the system components X and Y. Note that since the state variables x and y are not known at time t_{n+1} simultaneously for both components, some approximation of the exchanged variables $\tilde{x}_{n+1}$ and $\tilde{y}_{n+1}$ have to be considered (section Organization of computations). Here, we use constant interpolation, that is $\tilde{x}_{n+1} = \mathbf{x_n}$ and $\tilde{y}_{n+1} = \mathbf{y_n}$, for simplicity.

Let us choose $\mu = -1$, $\lambda = -2$, $a = 3$, $b = -3$ and $H = h$ for simplicity. Figure 1 shows the solution of the system (1) approximated by the backward Euler method with $h = 0.6$. Backward Euler is stable when applied to the system described by (2), that is when the system is solved as a whole. However the method shows unstable behavior when applied to each component in (3) separately and $H = h$.

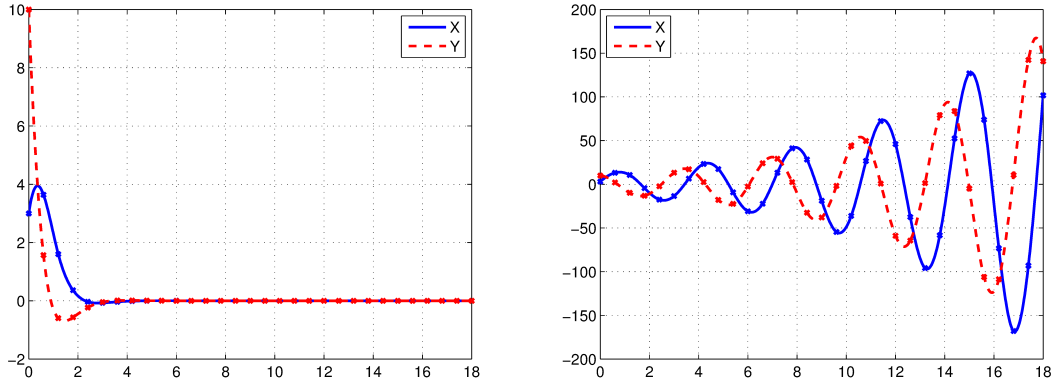

Figure 1. The backward Euler method is stable when applied to the whole system (A), but unstable when used to solve each component separately (B).

References

1. Strehmel K, Weiner R (1984) Partitioned adaptive Runge-Kutta methods and their stability. Numerische Mathematik 45: 283–300.
